# Brief Report: Characterization of Sensory Over-Responsivity in a Broad Neurodevelopmental Concern Cohort Using the Sensory Processing Three Dimensions (SP3D) Assessment

**DOI:** 10.1007/s10803-022-05747-0

**Published:** 2022-09-30

**Authors:** Maia C. Lazerwitz, Mikaela A. Rowe, Kaitlyn J. Trimarchi, Rafael D. Garcia, Robyn Chu, Mary C. Steele, Shalin Parekh, Jamie Wren-Jarvis, Ioanna Bourla, Ian Mark, Elysa J. Marco, Pratik Mukherjee

**Affiliations:** 1 Department of Radiology and Biomedical Imaging, University of California, San Francisco, San Francisco, CA USA; 2 Cortica Healthcare, San Rafael, CA USA; 3 Department of Psychology and Neuroscience, University of Colorado Boulder, Boulder, CO USA; 4 Growing Healthy Children Therapy Services, Rescue, CA USA

**Keywords:** Autism, ASD, Sensory Processing, Sensory Over-Responsivity, SP3D

## Abstract

Sensory Over-Responsivity (SOR) is an increasingly recognized challenge among children with neurodevelopmental concerns (NDC). To investigate, we characterized the incidence of auditory and tactile over-responsivity (AOR, TOR) among 82 children with NDC. We found that 70% of caregivers reported concern for their child’s sensory reactions. Direct assessment further revealed that 54% of the NDC population expressed AOR, TOR, or both – which persisted regardless of autism spectrum disorder (ASD) diagnosis. These findings support the high prevalence of SOR as well as its lack of specificity to ASD. Additionally, AOR is revealed to be over twice as prevalent as TOR. These conclusions present several avenues for further exploration, including deeper analysis of the neural mechanisms and genetic contributors to sensory processing challenges.

Children experience the complexities of the dynamic world around them through sensation. The perception and processing of sensory input lays the most integral foundation for their ability to integrate new experiences with what they already know and to continue to develop new thoughts and skills over a lifetime. Many children, though, react to typically non-noxious stimuli with aversion, under-awareness, and/or desire for increased stimulation – all of which create uniquely challenging experiences as these children strive to grow and align with their peers’ pace in reaching milestones. These difficulties present into three classifications – sensory over-responsivity (SOR), sensory under-responsivity (SUR), and sensory craving (SC), respectively – which comprise the phenotypic outcomes of sensory modulation disorders (Miller et al., 2021). Everyday experiences involve the integration of complex internal and external stimuli, including auditory and tactile information, and can present challenges in these and other sensory domains individually or in combination. As will be further explored through this study, non-noxious auditory and tactile stimuli are processed differently, thus leading to hyper-reactivity. This distress leads to challenges in several aspects of daily functioning, including emotional regulation (Ben-Sasson et al., 2009), anxiety (Green et al., 2012), academic ability (Ashburner et al., 2008; Dunn, 2001), sleep (Mazurek & Petroski, 2015), social behavior (Ben-Sasson et al., 2009), and motor skill performance (Liu, 2013). Sensory processing challenges have often been studied as a subset of autism spectrum disorders (ASD) (Crane et al., 2009; Kirby et al., 2022; Lane et al., 2010, 2011; Minshew & Hobson, 2008; Robertson & Baron-Cohen, 2017; Tavassoli et al., 2016; Wiggins et al., 2009) as they often co-occur with social communication challenges which are the hallmark of ASD. However, it is now understood that sensory processing challenges – and SOR specifically – can exist either as symptoms of ASD or independently (Chang et al., 2014). As far as we are aware, the prevalence of auditory and tactile over-responsivity (AOR, TOR) has not yet been explored in community clinics serving children with neurodevelopmental concerns (NDC) using an in-depth direct assessment of sensory processing (Boterberg & Warreyn, 2016; Cheung & Siu, 2009; Gourley et al., 2013; Feldman et al., 2020; Tomchek and Dunn, 2007). The prevalence of AOR and TOR has been analyzed through direct assessment within a slightly varied cohort from ours, and revealed that the rates of AOR (31%) and TOR (27%) were similar (Tavassoli et al., 2019). Other sensory assessment tools in research settings have suggested a range of prevalence of sensory processing challenges in individuals with ASD at rates from 50 to 95% (Baranek et al., 2006; Crane et al., 2009; Jussila et al., 2020; Klintwall et al., 2011; Lane et al., 2010; Leekam et al., 2007; Tavassoli et al., 2016; Wiggins et al., 2009). The aim of this study is to assess the prevalence of AOR and TOR in all comers to a community-based pediatric neurodevelopment center. We employ both a caregiver report measure of neurodevelopmental challenges, the ESSENCE-Q-REV (Gillberg 2012) and a direct assessment of sensory over-responsivity, the Sensory Processing Three Dimensions (SP3D) (Mulligan et al., 2019). We hypothesize that SOR is common in a general NDC cohort; that auditory and tactile over-responsivity are not exclusively secondary or co-morbid with other ASD symptoms; and that the rates of auditory and tactile over-responsivity are equivalent in this NDC cohort as a whole as well as in the subset with ASD.

## Methods

### Study Design

Children of 8 to 12 years of age are sequentially enrolled in a collaborative study between the University of California, San Francisco and Cortica Healthcare, a community-based pediatric neurodevelopment clinic and research center in Marin County, California. This is part of a larger observational cross-sectional study investigating the neural mechanisms of SOR approved by the UCSF Institutional Review Board (IRB# 19-27681). Informed consents and assents are obtained from caregivers and participants, respectively, in accordance with IRB policy.

### Participants

Study participants are recruited following medical intake at Cortica Healthcare. Each participant undergoes a thorough review of their history based on a standardized parent report, Cortica Neurodevelopment Intake Form, a general and neurodevelopmental physical examination, and record review by the Cortica physician and research coordinator. Exclusion from the study is based on the following criteria:

- Nonverbal Index ≤ 70 on the Wechsler Intelligence Scale for Children (Fifth Edition)*.
- Below ESSENCE-Q-REV “optimal cutoff” for neurodevelopmental concerns.
- Caregiver(s) unable to complete intake forms.
- In utero toxin exposure.
- Gestational age < 32 weeks or intrauterine growth restriction (birth weight < 1500g).
- Hearing or visual impairment.
- Additional medical/neurologic condition, including active epilepsy, malignancy, or known brain injury/malformation.

*A Nonverbal Index of less than or equal to 70 on the Wechsler Intelligence Scale for Children was selected to optimize the participants’ ability to tolerate the MRI included in the parent study.

### Developmental Measure

Children are screened for eligibility in part through the ESSENCE-Q-REV, a 12-question caregiver screener for ESSENCE disorders, including ASD, attention deficit hyperactivity disorder (ADHD), developmental coordination disorder, specific language impairment, and Tourette’s syndrome (Gillberg 2012). The response options are ‘No,’ ‘Maybe / A Little,’ or ‘Yes.’ The threshold for inclusion (“optimal cutoff”) for this measure is at least one ‘Yes’ or at least two ‘Maybe / A Little’ responses in total.

### Sensory Measure

This study employs direct sensory characterization by a licensed pediatric occupational therapist to investigate the prevalence of AOR and TOR in a pediatric NDC population. In order to determine SOR categorization, three auditory and two tactile probes from the SP3D are utilized. The auditory games include “Sounds & Pictures Matching Game” in which the participant matches sounds played to pictures displayed in front of them, “Orchestra Time” in which the examiner sets the beat of the music through clapping and then instructs the participant to play various instruments (cymbals, stick and cymbal, and a whistle) to the rhythm of an audio track, and “Find a Picture Game” in which the participant locates pictures in a booklet while the examiner plays an unrelated audio track. The tactile portion of the assessment includes the “Goo Game” in which the participant extracts a small plastic toy from a container of slime, and the “Painting Game” in which the participant is instructed to stroke a paintbrush and foot scrubber along their arm, followed by tracing their lips with a foam toothette. The participant is given a score of 1 (typical), 2 (mild/moderate), or 3 (severe) in reference to the intensity of their aversive reaction to each game. A score of 2 or 3 in any of the games corresponds to an SOR designation in the respective domain(s) and may be observed as grimacing during “Orchestra Time” or reluctance to interact with the slime in the “Goo Game,” for example. A participant, therefore, can be described as auditory over-responsive, tactile over-responsive, both, or neither.

### Autism Measure

Participants are further evaluated for a research designation of ASD first through the Social Communication Questionnaire (SCQ; Rutter et al., 2003) caregiver report form. Those scoring at or above a total score of 15 on the SCQ are then evaluated for ASD through the Autism Diagnostic Observation Schedule, Second Edition (ADOS-2; Lord et al., 2012). Individuals scoring above the ASD diagnostic cutoff on both the SCQ and the ADOS-2 are included within the ASD subgroup for the study. All others scoring below the diagnostic cutoff on the ADOS-2 or below 15 on the SCQ are assigned to the non-ASD subgroup.

### Statistical Analysis

Group characteristics were compared using a Student’s *t*-test for age, IQ composite scores, and parent education and income levels. Parental education levels were coded into a designated series of years of education (i.e. “High School or GED” is designated as 12 years and “College Graduate” is designated as 16 years) and only included responses from both biological parents. This resulted in a possible range of 16 to 40 combined years of education. Caregiver income levels were determined by totaling the midpoints of one or both primary caregivers’ income brackets and thus resulted in a possible range of $12,500 to $750,000 per year. Individuals who selected the “Prefer not to answer” option were treated the same as giving no response to the survey question. Further, two-tailed *z-*tests of proportions were used to compare ethnicity and race, while a chi-square test was used to analyze both parent questionnaire responses and sex differences between the two subgroups. A one-tailed, one-sample *z*-test of proportions was used to test the prevalence of SOR concerns, with “common” prevalence designated to be greater than 20%. Commonality was set at a threshold of 20% because neither ASD or ADHD have a prevalence close to surpassing this threshold (2.3% for ASD, 9.4% for ADHD) (Autism and Developmental Disability Monitoring (ADDM) Network, 2022; Data and Statistics about ADHD, 2021). A one-tailed *z*-test of proportions between two distinct populations was used to determine similarity between the proportions of AOR, TOR, or both in the ASD and non-ASD subgroups. An unpooled, one-tailed, two-sample *z*-test of proportions was performed to investigate the difference in prevalence of AOR and TOR in the NDC cohort. Significance was determined at a 95% confidence interval.

## Results

### Participants

Three-hundred and twenty-one children were enrolled into the medical practice, 132 individuals were excluded (Table1), 82 participants were enrolled (Tables2 and 3) with 107 individuals remaining in the screening and recruitment process. All participants had both ESSENCE-Q-REV and SP3D data for analysis.

**Table 1:** Exclusion Reasons and Totals (*n* = 132)

Reason	Count
*Aged out*	22
*NVI < 70*	29
*In-uterotoxinexposure*	6
*Premature*	11
*Visual impairment*	1
*Medical / neurologicalcondition*	47
*MRI incompatibility* ^*^	3
*Other* ^*†*^	13

*MRI incompatibility = cochlear implant, dental work, claustrophobia

†Other = moved from area, not approved by medical provider, and/or discontinued care at Cortica

**Table 2:** Demographic Characteristics of Included Participants by Cohort

Characteristic	NDC group *n*= 82	ASD subgroup *n*= 13	Non-ASD subgroup *n*= 66	*p-*value betweenASD and non-ASD subgroups
Mean age (years)	10.42	10.45	10.45	0.993
Sex (m,f)	60, 22	11, 2	47, 19	<0.001
Ethnicity(proportionHispanic orLatin American)	0.04	0	0.05	0.873
Race proportion White proportion Black / African American proportion Asian proportion “Other” proportion selected > 1 race	*n* = 79 0.81 0 0.04 0.01 0.14	*n* = 12 0.83 0 0 0 0.17	*n* = 65 0.80 0 0.05 0.02 0.14	0.789 **–** 0.448 0.665 0.798
Biological parent combined education level (average combined years)	34 *n* = 68	34.22 *n* = 9	33.89 *n* = 57	0.814
Primary caregiver combined income level (average midpoint yearly income total)	$313,194 *n* = 54	$383,928 *n = 7*	$312,222 *n = 45*	0.261

**Table 3:** Cognitive Metrics of Included Participants by Cohort

WISC-V Metric	NDC group *n*= 82	ASD subgroup *n*= 13	Non-ASD subgroup *n*= 66	*p-*value between ASD and non-ASD subgroups
Mean VCI	109.83	107.31	110.98	0.350
Mean VSI	108.91	109.08	109.42	0.895
Mean FRI	109.13	106.77	110.11	0.429
Mean WMI	97.46	92.46	99.55	0.179
Mean PSI	90.45	86.77	91.94	0.135

### ESSENCE-Q-REV

The ESSENCE-Q-REV revealed notably high caregiver concern for activity, attention/concentration, mood, sensory reactions, general development, and behavior within the NDC population. 85% of caregivers of the ASD subgroup exhibited some level of concern for their child’s sensory reactivity, and 65% of caregivers of the non-ASD subgroup exhibited concern in this realm. Overall, the distribution of proportions between the two subgroups was not significant for any of the questions of the ESSENCE-Q-REV (Table4).

**Table 4:** ESSENCE-Q RESULTS Percentages of responses of some level of concern (“Maybe/A Little” or “Yes”) to the first 11 questions of the Gillberg ESSENCE-Q-REV screening tool among children with neurodevelopmental concerns by ASD subgroup (*n* = 82)

ESSENCE-Q-REVQuestion	ASD subgroup (*n* = 13)	Non-ASD subgroup (*n* = 66)	*p*-value between ASD and non-ASD subgroups
General development	76.9%	66.7%	0.977
Motor development / milestones	69.2%	39.4%	0.870
Sensory reactions	84.6%	65.2%	0.983
Communications / language / babble	76.9%	47.0%	0.832
Activity or Impulsivity	92.3%	93.9%	1.000
Attention / Concentration / “Listening”	92.3%	92.4%	1.000
Social interaction / Interest in other children	92.3%	57.6%	0.975
Behaviour	76.9%	65.2%	0.967
Mood	76.9%	78.8%	0.999
Sleep	61.5%	56.1%	0.657
Feeding	61.5%	43.9%	0.657

### SP3D

**Fig. 1:**
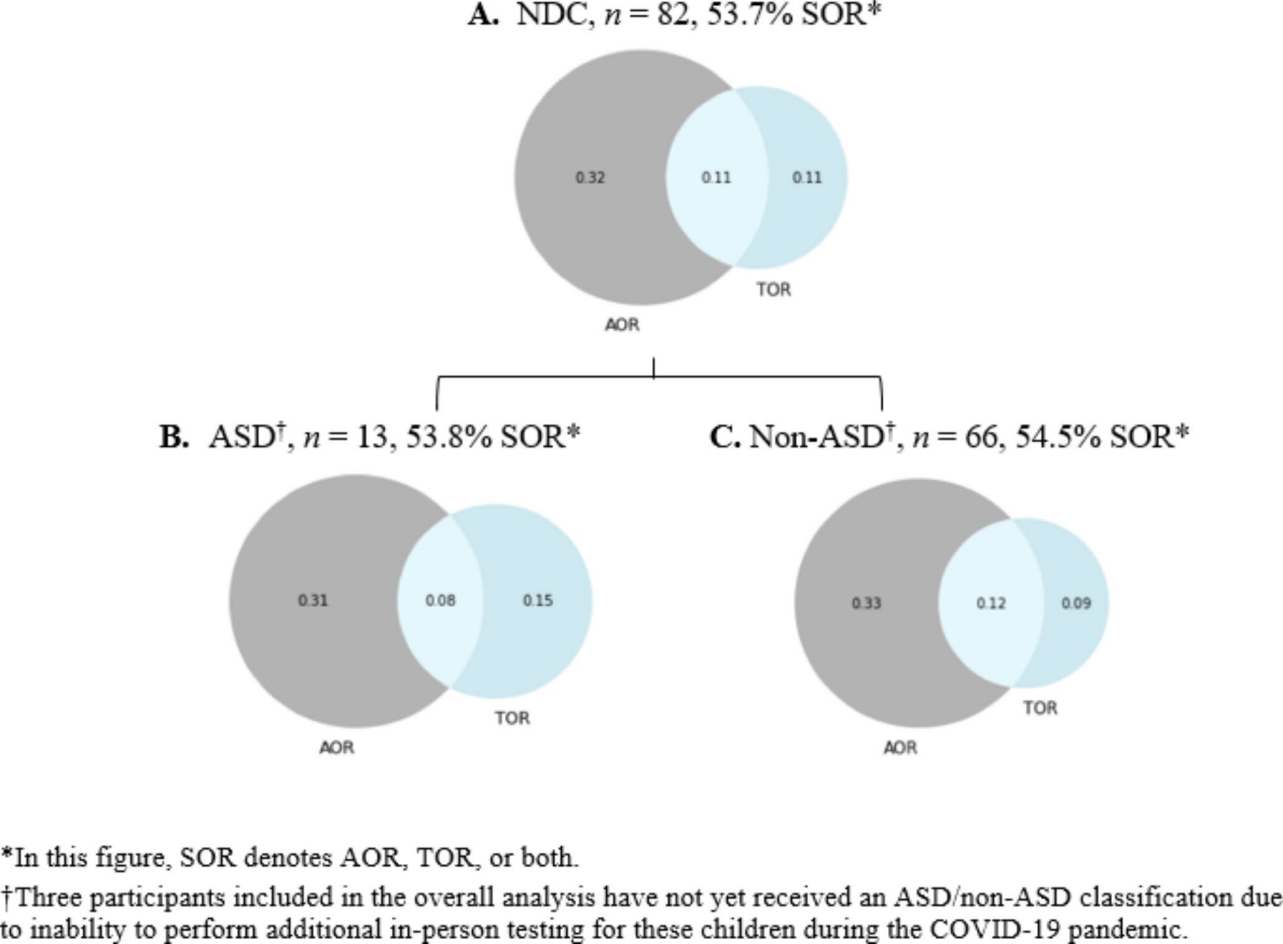
Proportions of Auditory and Tactile Sensory Over-Responsivity in Children by Cohort

Utilizing the SP3D assessment, 35/82 children (42.7%) meet criteria for AOR, 18/82 (22.0%) meet criteria for TOR and 9/82 (11.0%) meet criteria for both AOR and TOR. In total, 44/82 (53.7%) of the NDC population present with AOR, TOR or both (Fig.1A). Given this finding, we computed that the proportion of participants with AOR, TOR, or both was higher than the threshold of 20% to be considered “common” (*p* < .001). Further, the percentage of AOR (42.7%) and TOR (22.0%) within this population provided evidence against the hypothesized similarity in their rates of prevalence (*p* < .001).

Thirteen of 79 (16%) participants meet research criteria for ASD. Three participants included in the overall analysis have not yet received an ASD/non-ASD classification due to inability to perform additional in-person testing for these children during the COVID-19 pandemic. Of the ASD population enrolled, 5/13 (38.5%) meet criteria for AOR, 3/13 (23.1%) meet for TOR, 1/13 (7.7%) meet criteria for both AOR and TOR, and 7/13 (53.8%) meet criteria for AOR, TOR or both. Of the non-ASD population enrolled, 30/66 (45.5%) meet criteria for AOR, 14/66 (21.2%) meet criteria for TOR, 8/66 (12.1%) meet criteria for both AOR and TOR, and 36/66 (54.5%) meet criteria for AOR, TOR or both. Comparing the proportions of AOR, TOR, or both in the ASD and non-ASD subgroups, we found that the prevalence of this categorization in the ASD subgroup was similar to that of the non-ASD subgroup, supporting our hypothesis that AOR and TOR are not specific to ASD (*p* = .481).

## Discussion

This glance into a broader ongoing study on sensory processing emphasizes the prominence of SOR as a major concern in children with any NDC. Through the SP3D, our team was able to explore the frequency of SOR among children with and without an ASD diagnosis, as well as the difference in prevalence between auditory- and tactile-specific over-responsivity across a neurodiverse pediatric cohort.

### Sensory Reactions as a Parental Concern

In using the ESSENCE-Q-REV as a preliminary screening mechanism, we have ensured that the NDC population sample properly includes individuals with parental concern in the several realms encompassed within the survey. In turn, use of this measure revealed that nearly 70% of all caregivers of participating children showed concern for their child’s sensory reactivity. Further, since we observed a lack of significant difference in proportion of concern by question between the ASD and non-ASD subgroups, the notion of sensory reactivity being similarly prevalent across both groups is introduced. In a study performed by Ahn et al., (2004), of 703 kindergarteners from an inclusive public-school cohort completed Short Sensory Profile questionnaires, only 13.7% were reported to have sensory processing challenges. Additionally, Reilly et al., (2019) reported on ESSENCE-Q-REV results from children with epilepsy and non-epilepsy related neurodisability and found that 52% of parents of children with non-epilepsy related neurodisability and 48% of parents of children with epilepsy showed some degree of concern with their child’s sensory reactivity. In comparison of the non-NDC rates of sensory-related concerns in the kindergarten cohort and the slightly elevated rates among the non-epileptic cohort, our reported rate of concern across a representative community-based NDC group, as well as separated ASD designation, reveals a starkly higher prevalence of reported sensory-related challenges among this population.

### SOR in ASD and Non-ASD Populations

Past studies which employed the Sensory Profile as their sole sensory measure have concluded that sensory processing challenges are much more common in children with ASD than without (Tomchek and Dunn, 2007; Kientz & Dunn 1997; Cheung & Siu, 2009); and while this is certainly the case, the comparison to all comers with NDC has not been previously assessed with a direct assessment tool to the best of our knowledge. Our use of the SP3D has revealed that the occurrence of AOR, TOR, or both in children with ASD (54%) is concordant with the low end of the previously reported range of generalized sensory processing challenges in this population (50-95%) and is identical for participants with and without an additional diagnosis of ASD. It is to be noted that masking and/or regulation of internal behaviors occurs frequently, and may manifest as unobservable sensory adversities that are unable to be captured by the SP3D (Jorgenson, et al. 2020; Mandy 2019). When our cohort was evaluated with the parent report ESSENCE-Q-REV, the overall prevalence was higher at 70% (approximately 85% and 65% when split by ASD and non-ASD, respectively) which lies in the middle of the previously reported range. Nonetheless, a prevalence of 70% with parent report and 54% with direct assessment confirms the first hypothesis that sensory over-responsivity is a common concern and occurrence for children with NDC. Additionally, the respective proportions of individuals with AOR, TOR or both between our ASD and non-ASD samples are not significantly different, which supports our hypothesized non-specificity of ASD for SOR. Sensory challenges have been described as exclusively being an ASD symptom, but our data using the SP3D framework support both the hypothesized high prevalence of SOR in a broad NDC cohort as well as SOR being indiscriminately represented across individuals regardless of ASD diagnosis.

### AOR vs. TOR

In addition to the finding that AOR and TOR are common in children with NDC, we were further able to dissect the levels of AOR and TOR as independent modalities of sensory processing (Fig.1). We found auditory SOR to be over twice as common as tactile SOR in children with NDC (Fig.1A), which provides evidence to reject the hypothesis of similarity between the rates of AOR and TOR. Through a deeper analysis of the results of a comparative study conducted by Wiggins et al., (2009) in which the Sensory Profile was utilized to compare levels of sensory-specific sensitivities between children with ASD and with other developmental delays, we isolated the AOR-specific Sensory Profile items as designated in Tavassoli et al., (2019). Through this method, we observed that there was no significant difference between the two cohorts regarding the AOR-specific items of the Sensory Profile, which is in line with our results. Further, the Tavassoli et al., (2019) study explores the prevalence of AOR and TOR among ASD and neurodevelopmental disorder (NDD) cohorts. There are notable differences in the cohort assignment process of this NDD cohort from our NDC cohort, as the Tavassoli et al., (2019) study is composed of participants actively recruited based on their ASD and/or sensory processing disorder diagnoses. The NDC population described in this study represents a broad community-based collection of participating children not only with ASD or sensory processing challenges, but also with concerns regarding their general development, motor development, communication, activity, impulsivity, attention, concentration, social interaction, behavior, mood, sleep, and/or feeding. Given this distinction, the Tavassoli et al., (2019) study concluded that the observed rates of AOR and TOR within their two cohorts were similar by the SP3D and Sensory Profile. In respect of the differences in cohort designations between the two studies, this outcome elevates the importance of further research into broad NDC populations in order to elucidate the true prevalence of sensory processing challenges.

### Limitations

The primary limitation of this interim analysis is the small sample size, both overall, and more prominently within the ASD cohort. Given this, this report may represent an imprecise estimate of true rates of AOR and TOR in autistic populations. Further, while there is no significant age difference, there is a significantly male-skewed sex disparity between the ASD and non-ASD groups. In addition, white and non-Hispanic/Latin American individuals with high parental income and education levels constitute a significant proportion of this cohort, which has resulted in a lack of racial, ethnic and socioeconomic diversity among our sample of children with NDC (Table2). It is also to be acknowledged that although individuals with a nonverbal IQ below 70 are excluded in this report, they are still a relevant population to consider given that there is emerging evidence that sensory phenotypes vary by IQ (Ausderau et al., 2014). As this report serves as an interim analysis within a larger ongoing study, we are actively recruiting more participants and thus aiming to balance these factors for later analyses and would recommend replication of these findings in urban, suburban, and rural community settings.

### Further Exploration

There remains a paucity of peer-reviewed data for the SP3D and the ESSENCE-Q-REV with which to compare our results. There are several avenues of further research that this report surfaces, including a greater emphasis on the foundational neural architecture behind the complexities of both pediatric and adult sensory processing difficulties, as well as investigation into the genetic patterns among this population. Beyond both methods, three key factors are important to prioritize: (1) it is crucial to elevate the existence of sensory processing itself as a prevalent challenge beyond just that of a symptom of ASD, (2) to isolate and delve into the specific types of sensory processing (SOR, SUR, SC), and (3) to dissect these types into their corresponding sensory domains. Sensory processing challenges were certainly evident among this group; and, in fact, among the NDC population described, 54% expressed SOR across the auditory and/or tactile domains, while only 23% presented with a research designation of ASD. Further, several groups have studied the evidence of sensory sensitivity among groups of children with ASD and without, yet there does not seem to be an overarching focus on a single type of sensory processing, such as over-responsivity. By looking deeper into existing studies and pulling out the uniquely SOR aspects, we are able reveal similar findings throughout existing work. Delineating SOR (and respectively, SUR and SC) from the generalized “sensory processing” terminology is crucial in fine-tuning this field of research. While this study pulls auditory and tactile over-responsivity from the overarching “SOR,” further endeavors may consider pulling the remaining sensory domains from the specific sensory processing type. Lastly, both the assessment of auditory and tactile SOR in individuals with cognitive impairment as well as sensory adversities in conjunction with social-based tasks are certainly future directions worth exploring.

## Conclusion

In all, this glance into the weight of SOR in the realm of pediatric neurodevelopment provides a strong push in the direction of deeper analysis of its etiology, as well as clinical and therapeutic avenues for alleviating challenges resulting from SOR. With the sensory processing direct assessment revealing that 1 out of every 2 children seen at this community clinic experience auditory or tactile SOR, this is also a call to action for the evaluation of other forms of sensory processing challenges and other sensory domains, such as visual, vestibular and interoception. These findings serve as insight into the necessity of clinical evaluation of sensory challenges in clinical settings as well as robust delineation of the neural mechanisms and genetics of SOR both within and separate from ASD in our research endeavors. Our hope is that this brief report may serve as a foundation to continue building our understanding of sensory over-responsivity as it occurs in a real-world setting.
